# Speech Understanding with a New Implant Technology: A Comparative Study with a New Nonskin Penetrating Baha System

**DOI:** 10.1155/2014/416205

**Published:** 2014-07-23

**Authors:** Anja Kurz, Mark Flynn, Marco Caversaccio, Martin Kompis

**Affiliations:** ^1^Department of ENT, Head and Neck Surgery, Inselspital, University of Bern, 3010 Bern, Switzerland; ^2^Cochlear Bone Anchored Hearing Solutions, Mölnlycke, Sweden

## Abstract

*Objective.* To compare hearing and speech understanding between a new, nonskin penetrating Baha system (Baha Attract) to the current Baha system using a skin-penetrating abutment. *Methods.* Hearing and speech understanding were measured in 16 experienced Baha users. The transmission path via the abutment was compared to a simulated Baha Attract transmission path by attaching the implantable magnet to the abutment and then by adding a sample of artificial skin and the external parts of the Baha Attract system. Four different measurements were performed: bone conduction thresholds directly through the sound processor (BC Direct), aided sound field thresholds, aided speech understanding in quiet, and aided speech understanding in noise. *Results.* The simulated Baha Attract transmission path introduced an attenuation starting from approximately 5 dB at 1000 Hz, increasing to 20–25 dB above 6000 Hz. However, aided sound field threshold shows smaller differences and aided speech understanding in quiet and in noise does not differ significantly between the two transmission paths. *Conclusion.* The Baha Attract system transmission path introduces predominately high frequency attenuation. This attenuation can be partially compensated by adequate fitting of the speech processor. No significant decrease in speech understanding in either quiet or in noise was found.

## 1. Introduction

**Figure 1:**
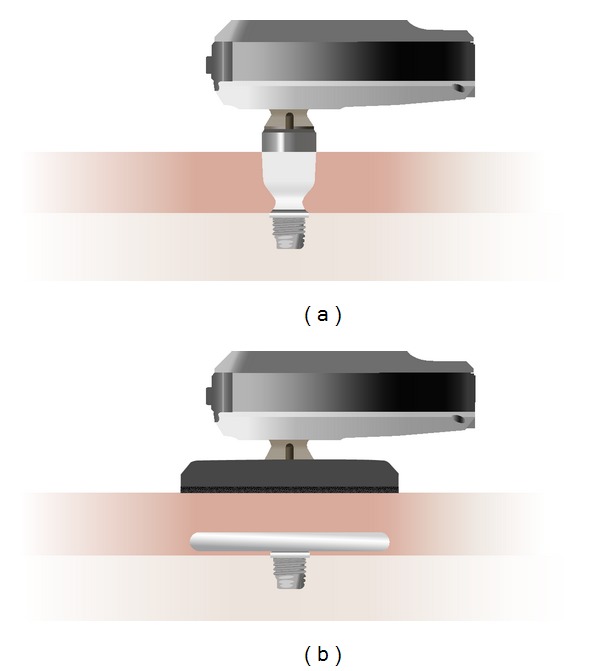
(a) Current Baha system with skin penetrating abutment. (b) Nonskin penetrating Baha Attract system with magnetic retention.

With more than 100,000 implantations so far, bone anchored hearing implants [1] belong to the widest used implantable hearing aids to date, second only to cochlear implants. The principle of operation is shown in Figure 1(a): a skin penetrating abutment is attached to an osseointegrated titanium implant. A sound processor is then attached to the abutment using a snap coupling which can be adjusted or removed by the user. Although each part of the system has been improved considerably in the last years [2–4], the basic design principle has now been in use for over 3 decades [5]. Its attractiveness is based on the relatively simple surgery and on the excellent results in adults and children with conductive or mixed hearing loss or, more recently, also in single sided deafness [6–8].

Despite this success, some drawbacks are well known. One of them is a tendency to low-grade infections around the abutment [9, 10], another personal preference, and cosmetic factors. Some patients who could benefit significantly from a system such as the Baha depicted in Figure 1(a) decline because of the skin penetrating implant behind the ear.

Several solutions, in which the skin remains intact, have been proposed. The Xomed Audiant system [11] in the 1980s had an implanted magnet, but the coil of the transducer was built into the sound processor. The maximal output of the system proved to be too low for numerous patients [11], and the system was discontinued. The Sophono system [12] is available today and is based on two implanted magnets within a single implant [13]. The sound processor with the bone conduction transducer (vibrator) is attached externally over the intact skin. The contact area between the skull and the implant is relatively large (more than 2.5 cm^2^) and new research suggests that its output is 10–15 dB lower than that of the Baha [14].

Recently, a new bone conduction implant, the Vibrant Bonebridge [15], has been introduced. In contrast to the other systems described so far, the transducer is fully implanted and connected to the speech processor via a radiofrequency link. The implant is significantly larger and more expensive than any of the others.

Very recently, a new system called Baha Attract has been proposed. It is shown schematically in Figure 1(b). It uses the same types of sound processors and the same osseointegrated titanium implant as the current Baha system. This results in a small contact area between the skull and the implant. Two magnetic discs are used: one with a diameter of 27.0 mm below the intact skin and another with a diameter of 29.5 mm, to which the external sound processor is attached. The choice of such a relatively large contact area results in lower skin pressures to achieve the necessary retention force.

So far, little is known about the practically important audiologic aspects of this new system and we are not aware of any peer reviewed reports or investigations. The change of the transmission path from direct bone conduction through the abutment to transmission via soft tissue might introduce differences in the acoustic transmission, most probably an additional attenuation. However, its extent and consequences for speech understanding are not known. A part of this attenuation may be compensated by different setting of the sound processor, but possibly not all of it.

The aim of this investigation is threefold. The primary aim is to compare the proposed Baha Attract transmission path to the conventional path shown in Figure 1(a) in actual Baha users. Hearing thresholds and speech understanding measured through the system are the pertinent endpoints.

The second aim is to estimate the hearing thresholds, at which candidates may be expected to experience significantly decreased speech understanding when deciding whether to choose a Baha Attract system instead of the skin penetrating solution.

The third aim is to ascertain that the differences in hearing between the two transmission paths are comparable for the two most important groups of Baha users: those with a conductive/mixed hearing loss [3] and those with single-sided sensorineural deafness (SSD) [8]. As all changes in the transmission path take place prior to sound entering the skull, we hypothesize that the attenuation and therefore the impact on speech understanding should be similar in both user groups. However, to date there is no experimental evidence to support this hypothesis.

## 2. Materials and Methods

### 2.1. Subjects

**Figure 2:**
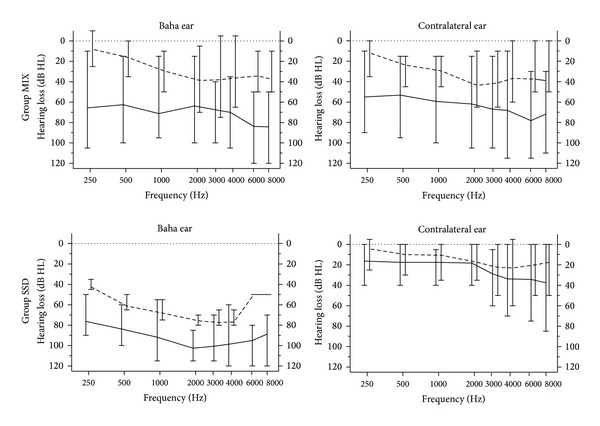
Pure tone audiograms of the two study subgroups MIX and SSD. Solid lines denote mean AC thresholds, broken lines mean BC thresholds, and error bars show the range.

16 adult Baha users aged 30–75, mean 58.4 years, 6 females and 10 males, participated in the study. All had an implant with a skin penetrating abutment, as shown in Figure 1(a), for a period between 6 months and 22 years (average 8.9 years, 7 right, 9 left) and used a Baha sound processor on a daily basis at the time of testing (6 Baha BP110, 5 Baha BP100, 3 Baha Intenso, 1 Baha Divino, and 1 Baha Compact [3, 4, 16]). Eight subjects had a mixed or conductive hearing loss and this group is labelled MIX throughout the text. The other 8 subjects had a single-sided sensorineural deafness. This group is labelled SSD throughout the text.  Figure 2 shows a synopsis of the unaided air conduction (AC) and bone conduction (BC) hearing thresholds for both groups.

### 2.2. Transmission Paths

**Figure 3:**
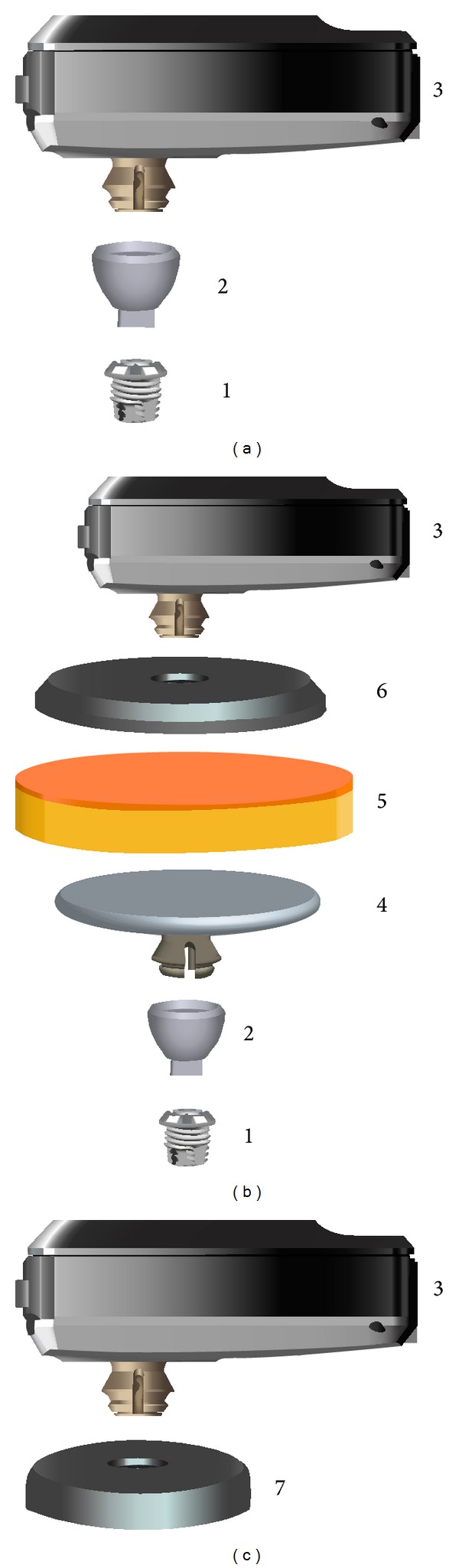
Transmission paths compared in the study. (a) “Abutment,” (b) “Magnet 3” and “Magnet 5,” and (c) “Testband” individual parts: (1) implant, (2) abutment, (3) sound processor BP110, (4) internal magnet, (5) artificial skin, (6) external magnet plate, and (7) testband (only the disc is shown, and the headband, which is attached to the disc, is not shown).

Four different transmission paths were compared in this study. They are labelled “Abutment,” “Magnet 3,” “Magnet 5,” and “Testband.” Figure 3 shows a schematic representation of these paths. A Baha BP110 sound processor [4] (Cochlear Inc., Sweden) was used for all tests and all participants.

In the “Abutment” setting, the BP110 sound processor was attached directly to the patient's own abutments, as shown in Figure 3(a).

In the “Magnet 3” and “Magnet 5” settings, shown in Figure 3(b), a magnetic plate (diameter 27.0 mm, thickness 2.4 mm) was attached to the snap coupling. An artificial skin sample (SawBones 1485-150, Sweden, diameter 28 mm, thickness 5.6 mm) was placed directly above it. The thickness of this sample is very close to the average skin thickness of 5.5 mm on the mastoid found by Faber et al. [10]. Then the Baha Attract sound processor magnet (diameter 29.5 mm, thickness 5.1 mm) was placed above the artificial skin sample and the BP110 sound processor was attached to its snap coupling.

Five different processor magnets are available, labelled 1 through 5 by the manufacturer according to their magnetic strength. In this investigation, Magnet 3 was used as it was the weakest one that was held in place sufficiently for the experiments without falling off. Its measured retention force in the experimental setup was 0.87 N.

In the “Magnet 5” setting, the strongest magnet available was used. Its retention force in the experimental setting was measured to be 1.24 N.

In the “Testband” setting, depicted schematically in Figure 3(c), the BP110 sound processor was mounted on a standard Baha testband [17]. The plastic disc of the testband, which holds the sound processor, is shown schematically in Figure 3(c). It was placed immediately behind the patient's implant, but without touching the abutment. The “Testband” condition was included because it is simple, is frequently used preoperatively at many centers, and is expected to show similar results as the Baha Attract transmission path.

### 2.3. Study Protocol

The study protocol was approved by the local Ethical Committee of Bern. All tests were performed at the University Hospital of Bern in accordance with the declaration of Helsinki and all participants had given their informed consent prior to inclusion in the study.

The measurements took approximately 5 hours per subject and were completed within one day for all but one participant. For each subject, AC and BC threshold were measured first in both ears. BC thresholds were measured between 250 Hz and 8000 Hz. Scale-out values were marked. They were rare, except for the poorer ear of the SSD group, as shown in the bottom left panel of Figure 2, where scale-out values were replaced by the audiometer limits for graphical purposes. Then unaided sound field thresholds were measured. For all sound field measurements, that is, aided and unaided, the ear contralateral to the Baha implant was plugged with an ear plug (E-A-Rsoft, 3M, Sweden) and covered with a Peltor Optime II hearing protector (Aero Ltd., Poynton, UK).

Then, hearing and speech understanding with each of the 4 transmission paths described in Section 2.2 were tested. The order of the transmission paths was varied systematically between subjects to minimize effects of training or fatigue.

For each new transmission path, first BC thresholds were measured directly via the BP110 sound processor (so-called BC Direct [18]). Then the processor was fitted according to the manufacturer's instructions, with the individually measured BC Direct values serving as the starting point. Version 2.0 SR 2 of the Cochlear Fitting software was used for all fittings. All automatic algorithms such as noise reduction or automatic directionality were switched off. For all tests, the everyday program was used with the following settings: microphone set to omnidirectional mode, feedback manager set to default, and position compensation set to “on.” No additional fine tuning was administered.

After an acclimatization period of 30 minutes, the following three measurements were performed in the sound field: (1) aided thresholds using narrow band noise, (2) aided speech understanding in quiet using German monosyllabic words (Freiburger word test) at a presentation level of 65 dB SPL, and (3) aided speech understanding in noise using the German Oldenburger sentence test (OLSA) [19]. The OLSA uses an adaptive test procedure to estimate the signal-to-noise ratio (SNR) required for 50% speech understanding. It consists of 40 lists of 30 test sentences each and an accompanying noise signal (speech babble) generated by superimposing all test items. The noise level was held constant at 65 dB SPL and the presentation level of the test sentences was varied adaptively according to the number of correctly repeated words, as prescribed by the predefined OLSA test paradigm [19]. Two training lists were administered before the actual testing. The results of the training lists were not used.

### 2.4. Test Rooms and Test Equipment

All measurements took place in a double-walled sound attenuating chamber (6.0 × 4.1 × 2.2 m) with an almost frequency independent average reverberation time of 0.14 s. Speech in quiet and sound field thresholds measurements were measured with a clinical audiometer (GSI61; Grason-Stadler, Mildford, NH, USA) using an active loudspeaker (Type 1030A, Genelec, Iisalmi, Finland) placed 1 m in front of the listener. For speech understanding in noise, an Audiobox amplifier (Merz Medizintechnik, Reutlingen, Germany) and a Control 1 Pro loudspeaker (JBL Ins., CA, USA) positioned at a distance of 1 m from the listener was used.

### 2.5. Statistical Analysis

Results were analyzed using Prism 5 and Instat 3.10 (both from GraphPad Inc., La Jolla, CA, USA). The Friedman test (repeated measures nonparametric ANOVA) and Dunn's comparisons as posttests were used for comparisons between the different transmission paths. Mann-Whitney tests were used for comparisons between the MIX and the SSD group in Section 3.4. All statistical analyses were either performed or supervised by a certified statistician (last author Martin Kompis).

## 3. Results

### 3.1. Hearing Thresholds through the Different Transmission Paths

Hearing thresholds were measured twice through each of the 4 transmission paths: once using BC Direct, that is, measuring the BC thresholds with the sound processor as the signal generator, and once as aided sound field thresholds, where the sound processor acts as a hearing amplifier in its clinically intended way. The most important difference between the two measures was that any additional damping in the transmission path may be compensated by suitably fitting the sound processor in the sound field measurement, but not in BC Direct measurement.

**Figure 4:**
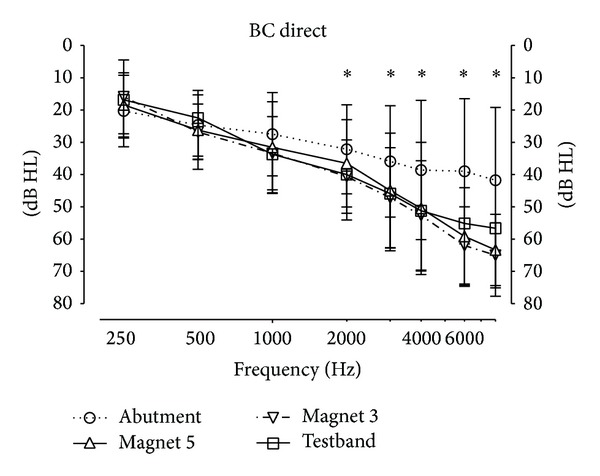
Hearing thresholds measured directly through the sound processor. Stars denote statistically significant difference (*P* < 0.05) between the 4 transmission paths at this frequency.

Figure 4 shows the BC Direct thresholds of all 16 participants. Below 1000 Hz, all thresholds are similar and no statistically significant differences are found (*P* > 0.05). Above 1000 Hz, the Friedman test shows significant differences (*P* ≤ 0.0072). The posttests reveal that the difference lies between the better threshold with the abutment and the other 3 transmission paths, but not between Magnet 3, Magnet 5, and the Testband. The difference between the abutment and the other 3 paths lies between 11.9 dB and 23.3 dB for the frequency range of 4 to 8 kHz.

**Figure 5:**
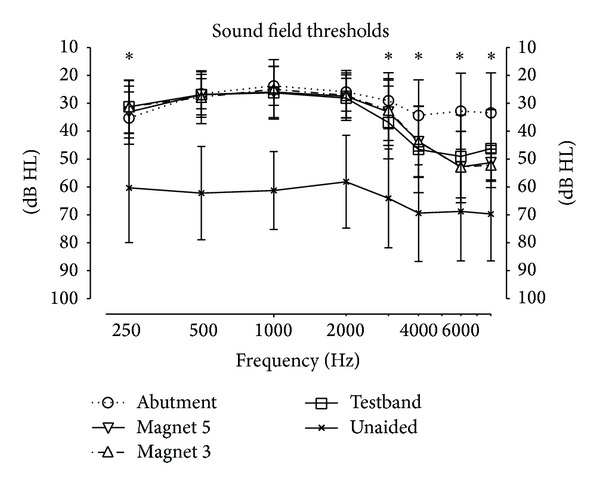
Unaided and aided sound field thresholds. Stars denote statistically significant difference (*P* < 0.05) between the 4 aided thresholds (4 transmission paths) at the given frequency.

Figure 5 shows the sound field thresholds in the unaided and in the aided condition using all 4 transmission paths. For all aided conditions and at all frequencies, the aided thresholds are significantly better than the unaided thresholds. Stars denote statistically significant differences (*P* < 0.05) between the aided conditions.

Aided thresholds are similar in the middle frequency range (500 to 2000 Hz) but differ at 250 Hz (*P* = 0.0016) and above 3000 Hz (*P* < 0.011). Again, the statistical posttests show that it is the difference between the abutment and the other conditions, and not between the 3 other transmission paths, which is statistically significant. In the frequency range 4000 to 8000 Hz, the difference between the abutment and the other 3 transmission paths is smaller by approximately 3 dB than that for the BC Direct measurement in Figure 4.

### 3.2. Aided Speech Understanding

**Figure 6:**
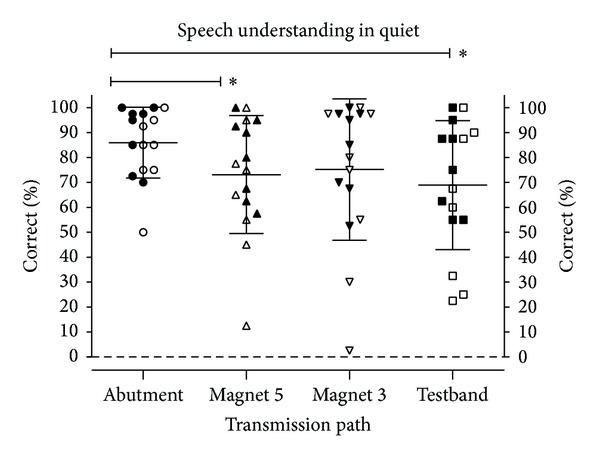
Monosyllabic word understanding in quiet at 65 dB SPL. Data points denote individual results (filled symbols: group SSD; empty symbols: group MIX), and horizontal lines denote mean values and standard deviations. Stars denote statistically significant differences (*P* < 0.05).

Figure 6 shows the results for aided speech understanding in quiet at a presentation level of 65 dB. Average scores decrease from the abutment setting to the testband (difference of 17% points) with the scores for “Magnet 3” and “Magnet 5” lying in between. Despite substantial variations between the participants, the differences are statistically significant (Friedman test *P* = 0.01). Dunn's posttests reveal that the difference between “Abutment” and “Magnet 5” as well as between “Abutment” and “Testband” is significant (*P* < 0.05). No significant difference was found between Magnet 3 and Magnet 5.

**Figure 7:**
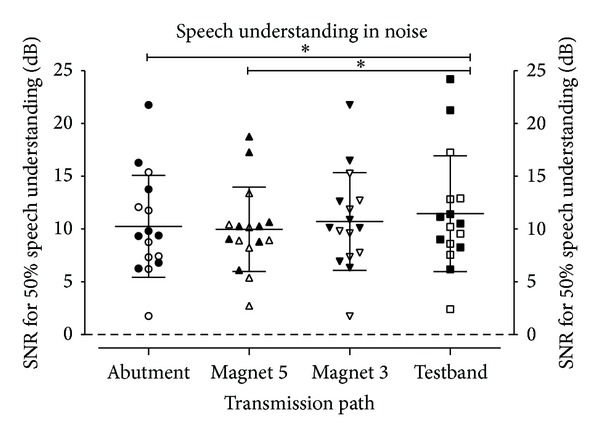
Speech understanding in noise. Lower SNRs denote better speech understanding. Data points denote individual results (filled symbols: group SSD; empty symbols: group MIX), and horizontal lines denote mean values and standard deviations. Stars denote statistically significant differences (*P* < 0.05).

Figure 7 shows the results for speech understanding in noise. Here, lower SNRs denote better speech understanding in noise. The differences between the means are small (max difference 2.0 dB) but still statistically significant (*P* = 0.02). The posttests show that the significant (*P* < 0.05) differences are between “Abutment” and “Testband” and between “Testband” and “Magnet 5.” Again, no significant difference was found between “Magnet 3” and “Magnet 5.”

### 3.3. Relationship between Unaided BC Thresholds and Speech Understanding

The attenuation introduced by the Baha Attract system can be partially compensated by proper adjustment of the sound processor. This can be seen by comparing Figures 4 and 5. Users with relatively good BC hearing thresholds can therefore be expected to experience only small or even no detrimental effect from using the Baha Attract system. In the higher frequency range, compensation is only partial (Figure 5). It is conceivable that mainly users with a more pronounced hearing loss might suffer a noticeable decrease in speech understanding when choosing a Baha Attract system instead of the current solution with the skin penetrating abutment.

**Figure 8:**
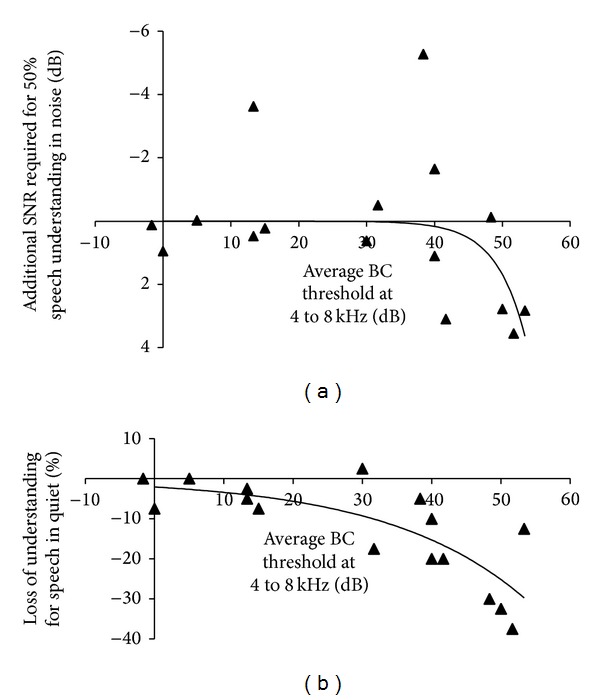
Difference between speech understanding in the “Abutment” and the “Magnet 3” condition as a function of the average BC hearing loss of the better ear at 4, 6, and 8 kHz. Data points for individual subjects and nonlinear regression lines are shown. (a) Speech understanding in noise. (b) Speech understanding in quiet. In both panels, data points closer to the bottom of the graph denote lower speech understanding with Magnet 3.

Aided thresholds and aided speech understanding are known to correlate well with the BC threshold of the better ear [20]. This correlation is better than the correlation with, for example, AC thresholds or with the BC threshold of the poorer ear [20]. The largest difference between the “Abutment” and the “Magnet” transmission paths shows in the high frequency region above 3000 Hz.  Figure 8 shows the loss of speech understanding when changing from the “Abutment” to the “Magnet 3” transmission as a function of the average BC thresholds at 4, 6, and 8 kHz. Scale-out was observed in 4 subjects at 6 or 8 kHz, never at 4 kHz. In cases of scale-out, audiometer limits were used. This may lead to a small compression at the right side of the graph in Figure 8 when compared to the real, but not readily measurable BC thresholds of these few subjects.

Both parts of the figure show the data points for each participant and an exponential (nonlinear) fit. There is a substantial spread of individual data points. Nevertheless, a tendency towards higher SNRs required for speech understanding in noise can be seen starting from around 40 dB average BC hearing loss. For speech in quiet (lower panel of Figure 8), there is a drop that similarly starts to be clinically significant around an average BC hearing loss of approximately 30 to 45 dB.

### 3.4. Single Sided Deafness versus Mixed Hearing Loss

So far, all data of the SSD and of the MIX group were pooled and analyzed together. This is based on the hypothesis, that the change in the transmission path should affect patients in both groups similarly, previously discussed.

To test whether this hypothesis can be substantiated by our data, the difference between the 2 groups of patients (MIX and SSD) was analyzed. For each of the 4 measurements (BC Direct, aided sound field thresholds, speech in quiet, and speech in noise), the difference between the “Abutment” and the “Magnet 3” setting was analyzed. Data for “Magnet 3” rather than for “Magnet 5” are shown here, as these differences might be expected to be larger due to the weaker coupling to the skin, although no significant differences between the two magnets were found in our data.

For the BC Direct thresholds over the 8 frequencies 250, 500, 1000, 2000, 3000, 4000, 6000, and 8000 Hz, the average difference between “Abutment” and “Magnet 3” ranges from −6.9 dB to +25.5 for the SSD group and between −1.9 dB and +26.3 dB for the MIX group. Even before correction for multiple testing, the difference between the two groups is not statistically significant at any of the 8 frequencies (Mann-Whitney test, *P* = 0.37 to 0.99).

Similarly, for aided sound field thresholds over the same 8 frequencies between 250 and 8000 Hz, the average difference between “Abutment” and “Magnet 3” ranges from −6.3 dB to +18.1 for the SSD group and between −1.9 dB and +23.1 dB for the MIX group. Again, even before correction for multiple testing, the difference between the two groups is not statistically significant at any of the 8 frequencies (Mann-Whitney test, *P* = 0.22 to 0.62).

Table 1 summarizes the comparison between the SSD group and the MIX group for speech in quiet and for speech in noise. Again, the differences between the MIX and the SSD groups are not statistically significant for either speech test.

## 4. Discussion

Our data suggest that the nonskin penetrating Baha Attract system should be helpful and beneficial for patients. Although on average speech understanding in quiet does decrease, if compared to the direct bone conduction through the abutment, the difference is small (10.8% points for Magnet 3 and 12.8% points for Magnet 5) and not statistically significant. The drop is even smaller than that for the “Testband” transmission path, which is used frequently by audiologists for preoperative testing. As a consequence, preoperative tests with a testband should be useful and valid predictors for the postoperative outcome with the Baha Attract system.

The additional attenuation of the Baha Attract system increases from around 5 dB at 1 kHz to 20–25 dB at 6 to 8 kHz, when compared to the abutment (Figure 4). In contrast, speech reception scores show only a relatively small drop. The probable reason for this difference is suggested by the aided sound field thresholds in Figure 5. As the sound processor was fitted for each transmission path separately, there is a good compensation of the additional attenuation for frequencies up to approximately 3000 Hz. For 4000 Hz to 8000 Hz, there is still a partial compensation. Thus a large portion of the frequency spectrum which is important for speech understanding remains almost unaffected. Consequently, speech understanding scores can be reasonably expected to remain high in Baha Attract users.

All tests were performed with two different magnets. No significant difference between the two magnets (3 and 5) was found in any of the tests (BC Direct, aided sound field thresholds, speech understanding in quiet, and speech understanding in noise; Figures 4 to 7). A probable reason for this finding is that the thickness of the artificial skin is not affected significantly by the pressure of the magnets. Young's modulus of the skin sample was estimated to be around 83000 N/m^2^ (±11%) from a series of simple measurements. The relatively low pressures of less than 2200 N/m^2^ even by Magnet 5 will therefore cause a compression of 0.15 mm or less. This small deformation does not change the density or elastic properties of the skin sample significantly.

This raises the following question: how realistic the experimental setup with the artificial skin depicted in Figure 2(b) is. Figure 4 shows the frequency dependent attenuation through the system, measured psychoacoustically using the BC Direct method. It can be seen that thresholds are not significantly different from the attenuation of the patients' own, real skin with the testband in the same figure. Similar frequency dependences and magnitudes of the sound attenuation of real skin in patients have also been published earlier by other groups (e.g., [17, 21]). This suggests that the artificial skin does indeed mimic real skin reasonably well.

It is known that skin thickness behind the ear does vary between patients [10], but different skin thicknesses were not compared in this investigation. The thickness of the artificial skin (5.6 mm) used in this study is close to the maximum thickness (6 mm) of soft tissue recommended by the manufacturer. For higher values, the manufacturer recommends soft tissue thinning. Preliminary trials with a double layer of the artificial skin, which would then correspond roughly to the upper limit of the skin thickness found on the mastoid [10], showed that even the strongest magnet currently available could not hold the sound processor in place sufficiently.

Our comparisons of the two patient groups (SSD and MIX) suggest that there are no significant differences in how hearing and speech understanding are affected by switching from a skin penetrating abutment to a nonskin penetrating Baha Attract transmission path. These findings confirm that it is admissible to group the data of the two populations for analysis. More importantly, however, they suggest that there is currently no reason to limit the Attract system to only one of these patient groups.

## 5. Conclusion

The nonskin penetrating Baha Attract system offers a new approach of partially implantable bone conduction hearing aids. In the preimplantation tests reported here, it was found that there is an additional attenuation, ranging from approximately 5 dB at 1000 Hz to 20–25 dB above 6000 kHz, when compared to the conventional transmission path using an abutment. However, aided sound field hearing thresholds show that a substantial part of this attenuation, mainly in the frequency range up to 3000 Hz, can be compensated by the individual fitting of the sound processor. This is a probable explanation for the relatively minor and statistically nonsignificant differences in speech understanding in quiet and in noise between the two different transmission paths. The loss in speech understanding is even smaller than that for the transmission through a testband, a method that is commonly used preoperatively to test the system. On the basis of this preimplantation trial, it can be reasonably expected that the nonskin penetrating Baha Attract system will be useful and beneficial for patients.

## Figures and Tables

**Table 1:** The difference (decrease) in speech understanding between the “Abutment” and the “Magnet 3” transmission paths is not statistically significantly different between the two subgroups SSD and MIX either in quiet or in noise.

Measurement	Group SSD (mean ± SD)	Group MIX (mean ± SD)	Difference between groups
Speech in quiet	15.0 ± 20.8%	6.6 ± 8.3%	8.4% (*P* = 0.75)
Speech in noise	−0.8 ± 1.8 dB	0.2 ± 2.9 dB	−1.0 dB (*P* = 0.80)
